# Neuroprotective Efficacy of a New Brain-Penetrating C-Abl Inhibitor in a Murine Parkinson’s Disease Model

**DOI:** 10.1371/journal.pone.0065129

**Published:** 2013-05-31

**Authors:** Syed Z. Imam, William Trickler, Shinya Kimura, Zbigniew K. Binienda, Merle G. Paule, William Slikker, Senlin Li, Robert A. Clark, Syed F. Ali

**Affiliations:** 1 Division of Neurotoxicology, US FDA/National Center for Toxicological Research, Jefferson, Arkansas, United States of America; 2 Department of Medicine, UT Health Science Center, San Antonio, Texas, United States of America; 3 Department of Geriatrics, University of Arkansas for Medical Sciences, Little Rock, Arkansas, United States of America; 4 Division of Hematology, Respiratory Medicine and Oncology, Department of Internal Medicine, Faculty of Medicine, Saga University, Saga, Japan; Baylor College of Medicine, Jiao Tong University School of Medicine, United States of America

## Abstract

Experimental evidence suggests that oxidative and nitrative mechanisms account for much of the dopaminergic neuronal injury in Parkinson’s disease (PD). The ubiquitously expressed non-receptor tyrosine kinase c-Abl is activated by oxidative stress and thus, may play a role in redox-mediated neurodegeneration. Recently, we reported that c-Abl is activated in PD and that a c-Abl inhibitor mitigated neuronal damage in a PD animal model, suggesting a novel neuroprotective therapeutic approach. In the studies presented here, we evaluated the efficacy of a potent and clinically relevant second-generation irreversible Abl kinase inhibitor, INNO-406, as a therapeutic agent for PD. Our studies reveal that INNO-406 is capable of preventing the progression of dopaminergic neuronal damage in a toxin-induced C57 mouse model of PD. Using bovine brain microvessel endothelium as an in vitro blood-brain barrier (BBB) model, we detected rapid and significant transfer of INNO-406. Additionally, pharmacokinetic analyses demonstrated significant nanomolar concentrations of INNO-406 in brain in the presence or absence of MPTP administration, however, INNO-406 did not alter the brain levels of MPP+ in MPTP-treated mice. Finally, we showed that 10 mg/kg of INNO-406 given to C57 mice for one week before MPTP treatment (4×20 mg/kg i.p., every 2 h) and then for one week after MPTP treatment decreased the loss of dopamine in the striatum by 45% and the loss of TH+ neurons in substantia nigra pars compacts by 40%. This treatment regimen also abrogated activation of c-Abl, tyrosine phosphorylation of the Abl substrate and E3-ubiquitin ligase parkin, and accumulation of the toxic parkin substrate AIMP2. We propose that compounds of the INNO-406 class of Abl inhibitors will be useful new neuroprotective drugs for the treatment of PD-like pathology in preclinical systems that should be easily translated to the clinic.

## Introduction

Parkinson’s disease (PD) is a devastating neurological illness that affects about 1–3% of the population older than sixty-five years [1], [2]. Dopaminergic neuronal loss of the substantia nigra is the pathological hallmark of PD. The cause and mechanisms underlying the loss of dopaminergic neurons in PD are poorly understood. A major barrier to the development of new and effective therapies for PD is the current limitation in understanding of the molecular and cellular events that lead to degeneration of the nigrostriatal dopamine system. The large majority of PD cases are sporadic, but in some patients parkinsonism is inherited [3]. Several gene loci are associated with familial PD.

Specific mutations in the *PARKIN* gene are associated with early-onset Parkinson’s disease (PD) [4], [5]. Oxidative, nitrative or nitrosative stress and dopaminergic stress are thought to impair the function of parkin through either covalent modifications and/or alterations in the solubility of parkin [6], [7], [8]. Oxidative and nitrative damage are also thought to be major mechanisms of dopaminergic neuronal injury, both in animal models of PD, and in human PD patients [8], [9]. The ubiquitously expressed non-receptor tyrosine kinase, c-Abl, is activated by oxidative stress [10], and thus activation of c-Abl may play a role in neurodegenerative disorders, wherein oxidative stress is one of the major pathological mechanisms. For instance in Alzheimer’s disease (AD), beta-amyloid (Aβ) activates c-Abl in hippocampal neurons [11], [12], and c-Abl levels are elevated in pre-tangle neurons in AD [12]. Inhibition of c-Abl activity with Imatinib (STI-571, imatinib mesylate or Gleevec, Novartis) protects hippocampal neurons from Aβ-induced apoptosis, and suppression of c-Abl mRNA levels protects NR2a cells from Aβ-induced toxicity [11]. Moreover, deregulation of proteasome function induces c-Abl-mediated cell death, thus linking c-Abl to the proteasome system [13]. Recently, we have identified the tyrosine phosphorylation of parkin by the oxidative stress-induced non-receptor tyrosine kinase c-Abl as a regulatory mechanism in parkin function [14]. Parkin is tyrosine phosphorylated in the N-terminal domain by c-Abl, and Imatinib, a specific c-Abl kinase inhibitor used for treating chronic myeloid leukemia and gastrointestinal stromal tumors, inhibits that tyrosine phosphorylation. Tyrosine phosphorylation of parkin results in impaired E3-ubiquitin ligase activity and auto-ubiquitination of parkin.

Imatinib, which is used in clinic as first line of treatment for chronic myeloid leukemia, is an effective c-Abl inhibitor and has a minimal capacity to cross blood-brain barrier (BBB), with an increase in the transport in the presence of radiation or P-glycoprotein (ABCB1) and breast cancer resistance protein [BCRP (also known as ABCG2)]-inhibitors [15]. However, it has not been shown to be effective in treating glioblastoma thus, opening a foray of development of second generation and brain permeable c-Abl inhibitors. One of the most selective and potent second-generation c-Abl inhibitor, INNO-406 (NS-187 or Bafetinib), has been shown to significantly enter brain and target glioblastoma [16].

Here, we show that INNO-406 carries the potential to slow down the progression of PD thus becoming a very suitable target molecule for the development as a potential therapy for PD. One major attraction of repositioning INNO-406 for PD therapy is the fact that this drug has already been through extensive phase I and II studies and now entering into phase III trials for different type of leukemia.

## Materials and Methods

### Neurotoxin Injections in Mice

All animal procedures were approved by and conformed to guidelines of Institutional Animal Care Committee (National Center for Toxicological Research, Jefferson, AR). Adult male C57BL/6 mice (n** = **8 per group) were pretreated for 1 week with daily 10 mg/kg INNO-406 (Innovive Pharma) or vehicle alone (25% DMSO/PBS) via intraperitoneal injection. On day 7 animals received four intraperitoneal injections of the neurotoxin, 1-methyl-4-phenyl-1,2,3,6-tetrahydropyridine (MPTP-HCl; 20 mg/kg free base; Research Biochemicals) in saline or saline alone at 2 h intervals. Daily INNO-406 injections continued up to one more week after the last injection of MPTP. Animals were sacrificed one week after last MPTP injection and processed and prepared for biochemical and neurochemical assessment as previously described [8].

For pharmacokinetic studies, INNO-406 was injected at 2.5 mg/kg/i.p. or 10 mg/kg/i.p. in C57 mice and brains were harvested 1 h, 4 h, and 24 h after a single injection. In another study, INNO-406 was injected at 2.5 mg/kg/i.p. or 10 mg/kg/i.p. in C57 mice in the presence or absence of MPTP (4×20 mg/kg/i.p. at 2 h interval) after last injection of MPTP and brains were harvested 1 h, 4 h, and 24 h after a single injection of INNO-406. Finally, brain levels of MPP+ in C57 mice were measured 1 h, 4 h, and 24 h after single injection of MPTP (60 mg/kg/i.p.) in the presence or absence of a single injection of INNO-406 (2.5 mg/kg/i.p. or 10 mg/kg/i.p.).

### Neuropathology and Neurochemistry

The mice were anesthetized with an overdose of ketamine HCl/xylazine HCl solution (Sigma, St. Louis, MO) and perfused transcardially with 10–20 ml phosphate-buffered saline (pH 7.4) followed by an equal volume of 4% paraformaldehyde in 0.1 mol/l phosphate buffer (pH 7.4). Brains were dissected out and post-fixed overnight in the same fixative at 4°C. The tissues were cryoprotected in sequential 10% (2 hours), 20% (2 hours), and 30% (overnight) solutions of sucrose, and then embedded in Tissue-Tek OCT compound (Sakura Finetek USA, Torrance, CA). Brains were processed for cryosectioning at 30 µm thickness in the coronal plane. Four series of slides containing every fourth section were prepared for substantia nigra, whereas six series of every sixth section were prepared for striatum. The standard avidin–biotin complex (ABC) method was employed to immunostain brain sections. Briefly, sections were treated with 1% bovine serum albumin in phosphate-buffered saline containing 0.3% Triton X-100 for 30 minutes and then incubated with rabbit anti-TH (Chemicon International, Billerica, MA) at 1∶2,000 dilution for 48 hours at 4**°**C. Sections were rinsed in phosphate-buffered saline and incubated with biotinylated goat anti-rabbit secondary antibody (1∶200) for 1 hour, followed by avidin–biotin peroxidase complex (ABC Elite Kit; Vector Laboratories, Burlingame, CA) at room temperature for 1 hour. The chromogen used was either 3-amino-9-ethyl carbazole (AEC Chromogen Kit; Sigma) or 3,3**′**-diaminobenzidine tetrahydrochloride (Liquid DAB Substrate Kit; Zymed, Carlsbad, CA). DAB-stained midbrain sections were counterstained with cresyl violet and used for stereology. The total number of Nissl+/TH-immunoreactive neurons in SNpc was estimated using the optical fractionator method in combination with unbiased counting rules, an approach that is not affected by either the volume of SNpc or the size of the neurons. Briefly, the reference space (SNpc) in each 30 µm thick midbrain section was outlined at Å∼10 magnification using Stereo Investigator workstation (MicroBrightField, Williston, VT) attached to an Axioplan 2 imaging microscope (Carl Zeiss), fitted with a DEI-750 CE video camera (Optronics, Goleta, CA) and a LEP MAC5000 motorized stage controller (Ludl Electronic Products, Hawthorne, NY). Anatomical landmarks were determined according to Paxinos and Franklin. Then at random start, Nissl+/TH-immunoreactive neurons were counted from every fourth serial section throughout the entire extent of the SNpc using a Å∼63 oil immersion objective (numerical aperture 1.4). Cells were counted only when their nuclei were optimally visualized, which occurred only in one focal plane. Optical densities (OD) of the TH+ fibers in the striatum were measured from digitized images of every sixth section using NIH ImageJ software (NIH, Bethesda, MD). Conditions for tissue processing, immunostaining, and image capturing were kept constant for all animals. The measurements were taken from dorsolateral aspects of the striatum that receive the majority of innervation from dopamine neurons of SNpc.

Relative OD of TH+ fibers in the striatum were calculated by subtracting the background OD from the measured OD of the dorsolateral aspects of the striatum.

The concentration of dopamine was determined in striatal tissues by a modified HPLC method combined with electrochemical detection [17]. Tissues were weighed in a measured volume (20% w/v) of 0.2 M perchloric acid containing the internal standard 3,4-dihydroxybenzylamine 100 ng/ml. The tissues were disrupted by ultrasonication and centrifuged at 4°C (15,000×g; 7 min), and 150 µl of the supernatant was removed and filtered through a Nylon-66 microfilter (pore size 0.2 µm; MF-1 centrifugal filter; Bioanalytical Systems). Aliquots of 25 µl representing 2.5 mg of brain tissue were injected directly onto the HPLC/ECD system for separation of analytes. The amount of dopamine was calculated using standard curves that were generated by determining in triplicate the ratio between three different known amounts of the amine and a constant amount of internal standard.

To assess if MPTP metabolism is altered due to c-Abl inhibition by INNO-406, HPLC with UV detection (wavelength 295 nm) was used to measure striatal MPP^+^ levels [18]. Mice treated as described above were sacrificed, striata were dissected out, immediately frozen, and stored at −80°C until analysis. On the day of the assay, striata were prepared for MPP^+^ measurements by sonicating the tissue samples in 5 vol (wt/vol) of 5% trichloroacetic acid containing 5 µg/ml of 4-phenylpyridine (Sigma) as internal standard. After centrifugation, a 50–100 µl aliquot of supernatant was injected onto a Hydrosphere C18 column (YMC Inc. Waters Corp.). The mobile phase consisted of 90% 0.1 M acetic acid/75 mM triethylamine (pH 2.35 adjusted with formic acid) solution and 10% acetonitrile. The flow rate was 1.2 ml/min. Concentrations of MPP^+^ were expressed as nanomol per gram of wet tissue.

### Cell isolation and Culturing of Primary Brain Microvessel Endothelial Cells (BBMEC)

The BBMEC were isolated by a modified method previously described [19]. Briefly, the meninges were removed from the gray matter of fresh cow cerebral cortices and mechanically homogenized through a 100 µm mesh screen. Dispase II enzyme (5 mg/ml) reconstituted in MEM isolation media (MEM (1 M), HEPES (50 mM), polymyxin B (50 ug/ml), gentamicin (50 ug/ml) and amphotercin B (2.5 ug/ml) at pH 7.4) was added to the freshly homogenized cerebral cortical tissue (10 mg/g brain tissue) and MEM isolation media at pH 9.0 was added (equal w/w of brain tissue). The mixture was placed in an incubated shaker for one hour (37°C, 150 rpm). Following this incubation, the supernatant was removed by centrifugation (10 min at 1570 rcf). The crude capillaries were isolated by re-suspending the cerebral cortical tissue in a solution (13% (w/v) dextran supplemented with MEM (1 M), HEPES (50 mM), gentamicin (40 µg/ml), polymyxin B (50 µg/ml) and amphotercin B (2.5 µg/ml)) followed by centrifugation (10 min at 9170 rcf). The crude capillary pellet was collected and re-suspended in a collagenase/dispase (5 mg/ml) solution at a final concentration of 0.1% (v/w) and placed in an incubated shaker (37°C, 150 rpm) for one hour. During this time a percoll gradient (50% final concentration, supplemented with MEM (1 M), HEPES (50 mM), gentamicin (40 µg/ml), polymixin B (50 µg/ml) and amphotercin B (2.5 µg/ml)) was set up by centrifugation (60 mins at 39200 rcf). The digested capillaries were centrifuged (10 min, 1700 rcf) to remove the enzymatic treatment and re-suspended in isolation media and pipetted into the percoll gradient tubes. The BBMEC cells were then separated by centrifugation (10 min at 1700 rcf). The freshly isolated BBMEC were extracted from the percoll gradient (3 cc syringe), pelleted, and re-suspended in BBMEC complete media [(45% (v/v) Minimal Essential Media, 45% (v/v) Ham’s F-12 nutrient mix, supplemented with 10 mM HEPES, 13 mM sodium bicarbonate, 50 µg/ml gentamicin, 10% (v/v) equine serum, 2.5 µg/ml amphotericin B, and 100 µg/ml sodium heparin)]. The BBMEC were plated (approximately 50,000 cells/cm^2^ density) on collagen-coated, fibronectin-treated culture plates, and incubated in a humidified incubator (37°C with 5% (v/v) CO_2_). The cells were used after reaching confluence, typically 8–12 days.

### Rapid Determination of Drug Analogues by Reverse Phase HPLC

The concentrations of both drugs, Imatinib and INNO-406, were analyzed by a rapid HPLC separation and UV-VIS detection. Briefly, the isocratic HPLC analysis for both Imatinib and INNO-406 were achieved on a C18 Zorbax column (150×4.6 mm, 5 um) (Phenomenex, Torrance, CA) with a simple mobile phase consisting of methanol and deionized water (50∶50% v/v/) at a flow rate of 1.0 ml/min. The effluents were monitored at 223 nm and quantified using the area under the peak from standard solutions dissolved in assay II buffer (0.4 to 25 µg/ml). Assay II buffer consisted of sodium chloride (122 mM), potassium chloride (3 mM), calcium chloride (1.4 mM), magnesium sulfate (1.2 mM), sodium bicarbonate (25 mM), HEPES (10 mM), d-glucose (10 mM) and potassium phosphate (0.4 mM) at Ph of 7.4.

### Directional Permeability Comparison of Drug Analogues in Primary BBMEC

The directional comparison of drug analogues transported in primary BBMEC monolayers was determined. These methods have been previously described [20], [21]. Briefly, the freshly isolated BBMEC were plated in triplicates (50,000 cells/cm^2^ seeding density) on collagen-coated, fibronectin-treated polycarbonate membrane inserts, and cultured until confluence (12 mm; 0.4 µm pore size, Costar Transwell™ 12-well, Cambridge, MA). Complete culture media from confluent BBMEC monolayers was replaced 24-hours prior to the start of the experiment. The confluent monolayers were mounted in side-by-side diffusion chambers (Peremegear) either the apical or the basolateral sides were supplied with fresh assay II buffer with drug (donor chamber, 10 µM). Samples (100 µl) were removed from the reciever compartment at various time points (0–90 min) and replaced with fresh buffer. The concentration of drug in the samples was determined spectrophotometrically by HPLC methodology reported above (232 nm wavelength). The directional transport was expressed as the percent of the respective drug across the BBMEC monolayers over time (Mean ± SD, n = 3). The apparent permeability coefficient was calculated as previously described using the following equation (1) [22].
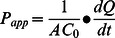
(1)Where, dQ/dt is the flux across the cell monolayers, A is the surface area of the membrane and C_0_ is the initial concentration of drug.

### Lactate Dehydrogenase Assay

The release of lactate dehydrogenase for cytotoxicity was measured by LDH assay kit (Roche#11644793001). A 100 µL of the supernatant from treated cells was transferred to new 96-well plates. The plates were incubated for 30 min at room temperature on orbital shaker. A colorimetric absorbance was recorded at 490 nm with a reference wavelength of 650 nm using a micro plate reader for colorimetric detection (BioTek, USA). Each experiment was done in triplicate. Cytotoxicity is expressed in terms of percent relative to the basal lactate dehydrogenase release by untreated control cells where controls cells serve as 100 percent.

### Statistical Analysis

Data analysis was performed with GraphPad InStat (GraphPad Software, La Jolla, CA) or Minitab 15 (Minitab, State College, PA). Statistical significance was determined using Student’s **t**-test or one-way analysis of variance at the 95% confidence level and was followed by pairwise multiple comparison tests (Tukey–Kramer multiple comparison test). Results are expressed as mean **±** SEM and considered significant at **P**<0.05.

## Results

### Permeability of INNO-406 and Imatinib in Primary BBMEC

**Figure 1:**
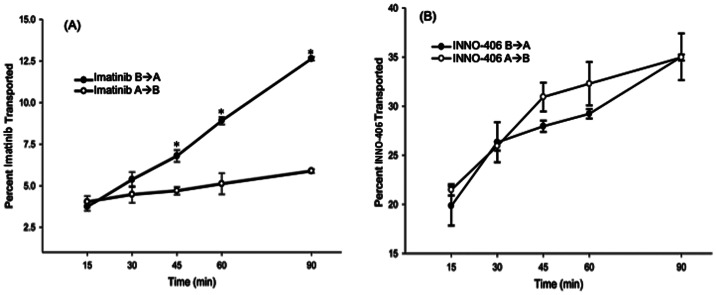
Directional transport of Imatinib (A) and INNO-406 (B) across BBMEC monolayers. The directional transport of two drug analogues Imatinib (A) and INNO-406 (B) were determined in BBMEC. The apical-to-basolateral transport (A→B) (open circles) and basolateral-to-apical (B→A)(closed circles). The data are presented as percent transported as a function of time (means ± SD, n = 3). * Considered statistically significant (p<0.05).

The trans-endothelial electrical resistance (TEER) results show the BBMEC monolayers have similar observed measures of resistance indicating the monolayers have similar barrier integrities with respect to tight-junction development (Table 1). The permeability results clearly demonstrate that INNO-406 has significantly enhanced (approximately 7.5-fold) apical-to-basolateral transport across the BBMEC monolayers when compared to Imatinib (Figures 1A and 1B). However, this enhanced transport is significantly reduced (2.5 fold) when comparing the basolateral-to-apical transport (Figures 1A and 1B). Additionally, Imatinib clearly demonstrates significantly increased directional transport in the basolateral-to-apical direction (Figure 1A), and this difference is absent for INNO-406 (Figure 1B). These observations are clearly demonstrated when comparing the apparent permeability of the drug analogues (Table 2). The observed apparent permeability coefficient for Imatinib shows significantly increased transport (nearly 5-times) from the basolateral side of the BBMEC monolayer when compared to the apical side (Table 2). These observations suggest the directional transport of Imatinib has different mechanisms. On the other hand, the observed permeability coefficients for INNO-406 clearly demonstrate equal transport in both directions across the BBMEC monolayers, and significantly greater when compared to INNO-406 (Table 2).

**Table 1:** BBMEC Trans-Endothelial Electrical Resistance (TEER).

Treatment Groups	TEER (ohms/cm^2^)	
	A → B	B → A
Imatinib	287±59	316±48
INNO-406	245±27	308±32

The data is presented as mean ± SD, n = 3.

**Table 2:** Directional Apparent Permeability Comparison of Drug Analogues.

Treatment Groups	Apparent Permeability Coefficient (1×10^−6^ cm/sec)		Apparent Permeability Ratio	
	A → B	B → A	B → A/A→ B	A → B/B→ A
Imatinib	12.96±0.070	63.86±0.230^*^	4.92±0.006^*^	0.20±0.001^*^
INNO-406	97.98±6.741	94.45±10.087	1.06±0.082	1.00±0.119

Where, dQ/dt is the flux across the cell monolayers, A is the surface area of the membrane and C_0_ is the initial concentration of drug. The data is presented as mean ± SD, and * considered statistically different directionally p<0.05, n = 3.

### Pharmacokinetics of INNO-406

**Figure 2:**
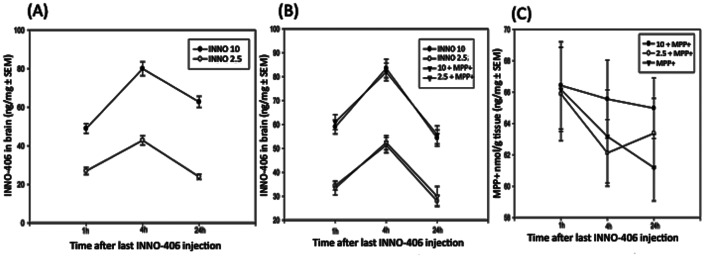
Kinetics of INNO-406 in mouse brain. Brain levels of INNO-406 (2.5 mg/kg/i.p. or 10 mg/kg/i.p.) in C57 mice 1 h, 4 h, and 24 h after a single injection (A). Brain levels of INNO-406 (2.5 mg/kg/i.p. or 10 mg/kg/i.p. after last injection of MPTP) in C57 mice 1 h, 4 h, and 24 h after single injection in the presence or absence of MPTP (4×20 mg/kg/i.p. at 2 h interval) (B). Brain levels of MPP+ in C57 mice 1 h, 4 h, and 24 h after single injection of MPTP (60 mg/kg/i.p. single injection) in the presence or absence of a single injection of INNO-406 (2.5 mg/kg/i.p. or 10 mg/kg/i.p.) (C).

To evaluate the bioavailability of INNO-406 in brain, we performed a time course analysis of INNO-406 in mouse brain in the presence or absence of MPTP. First, we evaluated brain levels of INNO-406 (2.5 mg/kg/i.p. or 10 mg/kg/i.p.) in C57 mice 1 h, 4 h, and 24 h after a single injection. Significant levels of INNO-406 were observed in mouse brain at 1 (50 ng), 4 (80 ng) and 24 (65 ng) h after single injection of 10 mg/kg INNO-406 (Figure 2A). These brain levels of INNO-406 were not altered by multiple MPTP injections (Figure 2B). Additionally, we evaluated the effect of INNO-406 on the bioavailability of MPP+. There was no significant alteration in the brain levels of MPP+ in C57 mice 1 h, 4 h, and 24 h after single injection of MPTP (60 mg/kg/i.p. single injection) in the presence or absence of a single injection (2.5 mg/kg/i.p. or 10 mg/kg/i.p.) of INNO-406 (Figure 2C).

### The c-Abl Inhibitor INNO-406 Protects against MPTP-induced Dopaminergic Damage

**Figure 3:**
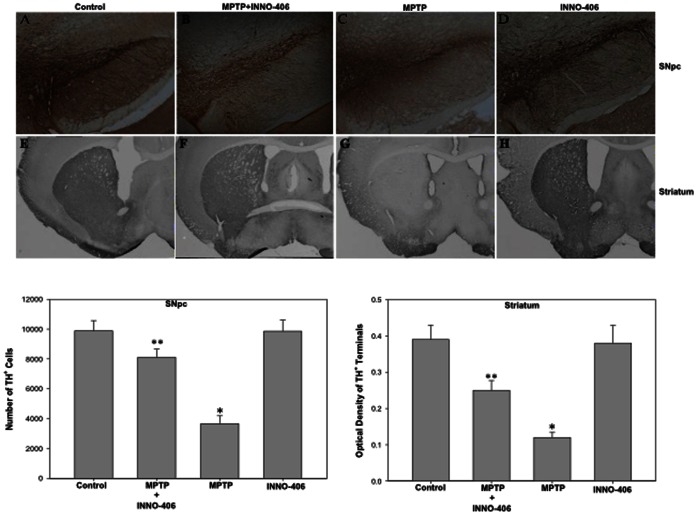
The c-Abl inhibitor INNO-406 protects against MPTP-induced loss of DA neurons. INNO-406 (10 mg/kg/i.p for one week prior to MPTP, 4×20 mg, and one week after MPTP treatment) protects (A–D) substantia nigra DA neurons and (E–H) striatal DA terminals from MPTP-induced toxicity. Pre-treatment of mice with INNO-406 leads to preservation of TH+ positive staining cells within the substantia nigra pars compacta and DA terminals in the striatum compared to MPTP treatment alone. Plots of quantitative stereological data showing the protective effect of INNO-406 in SNpc and striatum has been shown. * Considered statistically significant from control (p<0.05). ** Considered statistically significant from MPTP (p<0.05). SNpc = Substantia Nigra *pars compacta*.

**Figure 4:**
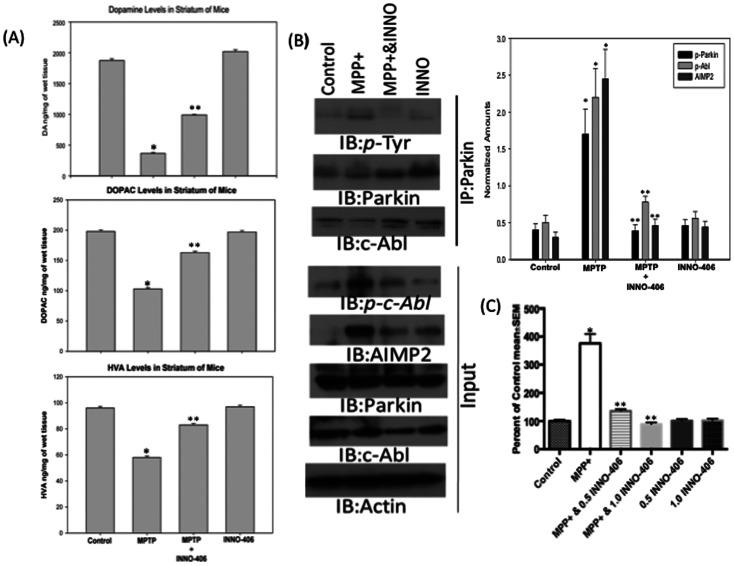
INNO-406 prevents dopaminergic depletion and c-Abl mediated parkin phosphorylation in mice striatum. (A) INNO-406 prevents MPTP-induced depletion of striatal dopamine and its metabolites DOPAC and HVA in adult male C57BL/J6 mice. Animals received INNO-406 (10 mg/kg, i.p.) as a single daily injection for 7 days before MPTP injection. On day 7 mice were treated with 4×20 mg/kg, i.p. of MPTP at 2 h interval. INNO-406 injection was continued for one more week after the last injection of MPTP. * Considered statistically significant from control (p<0.05). ** Considered statistically significant from MPTP (p<0.05). (B) Parkin is tyrosine phosphorylated in the striatal lysates of MPTP-treated mice. Animals received INNO-406 (10 mg/kg, i.p.) as a single daily injection for 7 days before MPTP injection. On day 7 mice were treated with 4×20 mg/kg, i.p. of MPTP at 2 h interval. INNO-406 injection was continued for one more week after the last injection of MPTP. Samples were immunoprecipitated with anti-parkin antibody and were immunoblotted for anti-*p*-tyrosine, anti-parkin or anti-c-Abl. A 10% input samples were immunoblotted for anti-*p*-c-Abl, anti-parkin, anti-AIMP2, anti-c-Abl and ant-Actin. A pretreatment with INNO-406, a c-Abl kinase inhibitor, blocks oxidative stress-mediated tyrosine phosphorykation of parkin, activation of c-Abl and accumulation of toxic substrate, AIMP2, in the striatum of MPTP-treated mice. The photomicrograph shown is a representative of three repeats from samples pooled from 3 different animals. The optical density quantification data as normalized amounts is presented. * Considered statistically significant from control (p<0.05). ** Considered statistically significant from MPTP (p<0.05). (C) Cytotoxicity plotted as a percentage of control as measured by LDH in SH-SY5Y cells treated with MPP^+^ (500 µM). Some samples were incubated with 0.5 or 1.0 µM INNO-406 for 6 h before MPP^+^ treatment. *p<0.05. Differences among means were analyzed using one-way analysis of variance (ANOVA). All experiments were repeated at least three times and representative examples are presented.

One week after the last MPTP or saline injection, mice were killed, and the neuroprotective effect of INNO-406 on the nigrostriatal dopaminergic system was assessed by quantitative analysis of TH+ neurons in the substantia nigra pars compacta (SNpc), as well as the density of TH+ terminals in the striatum. The organization and intensity of TH-immunoreactive neurons were essentially similar in the saline-treated and INNO-406 animal groups (Figure 3A, D). Stereological analysis demonstrated up to a 60–65% loss of TH+ neurons in the SNpc of mice following MPTP treatment, compared with saline-treated animals (Figure 3C). In contrast, there was only a 15–20% MPTP-induced loss of TH+ neurons in the SNpc of mice that were also treated with INNO-406 (Figure 3B). Parallel results were observed for striatal dopamine fiber terminals. In order to quantify the intensity of TH staining, optical density measurements were performed on the dorsolateral aspects of the striatum, which receive the largest share of innervation from dopamine neurons of the SNpc. By this method, TH immunoreactivity within the striatum was similar between control and INNO-406 mice (Figure 3E, H). Relative to the controls, there was an average 70% loss of the TH staining intensity in mice with MPTP treatment (Figure 3G), whereas the loss in mice that also received INNO-406 was only 35% (Figure 3F). To further confirm the neurochemical correlate of our findings, tissue levels of dopamine were determined biochemically. We show that 10 mg/kg of INNO-406 given to C57 mice for one week before MPTP treatment (4×20 mg/kg i.p., every 2 h) and then for one week after MPTP treatment prevented the loss of DA and its metabolites DOPAC and HVA in the striatum by 45% (Figure 4A).

### INNO-406 Prevents MPTP-induced Parkin Phosphorylation, Substrate Accumulation and Cellular Toxicity

The potent parkinsonian neurotoxin, MPTP (4 doses of 20 mg/kg i.p. every two hours) intoxication of mice led to substantial c-Abl activation 7 days after last dose of MPTP, as indicated by increased striatal levels of phospho-c-Abl (*p-c-Abl*), tyrosine phospho-parkin (*p*-Tyr), and AIMP2 (Figure 4B). INNO-406 (10 mg/kg i.p. daily for one week before and one week after MPTP) treatment resulted in protection against MPTP-induced injury, as reflected by significant decreases in striatal levels of phospho-c-Abl, phospho-parkin, and AIMP2 (Figure 4B).

Additionally, INNO-406 treatment prevented MPP+-induced cellular toxicity in SHSY-5Y neuroblastoma cells suggesting that our *in vivo* observation of INNO-406 prevention of parkin phosphorylation and AIMP2 accumulation might play a major role in cell survival (Figure 4C). A 6 hours pretreatment of SHSY cells with either 0.5 or 1.0 µM of INNO-406 prevented MPP+-induced (500 µM) cytotoxicity 24 hours after the exposure of these cells to MPP+. These data provide significant evidence that INNO-406 mediated prevention of parkin’s loss of function might afford a cell survival effect during the progression of PD.

## Discussion

The c-Abl tyrosine kinase participates in a variety of cellular functions, including regulation of the actin cytoskeleton, regulation of the cell cycle, and the apoptotic/cell cycle arrest response to stress, and the Abl family of kinases has been shown to play a crucial role in development of the central nervous system [23]. Recent studies have shown c-Abl activation in human Alzheimer’s and Parkinson’s diseases and c-Abl activation in mouse models and neuronal culture in response to amyloid beta fibrils and oxidative stress [11], [14].

Recently, we reported that oxidative and dopamine stresses lead to c-Abl activation, parkin tyrosine phosphorylation, and the consequent loss of parkin ubiquitination-dependent cytoprotective function [14]. c-Abl-mediated parkin inactivation in response to oxidative and dopaminergic stress seems to be the dominant pathway induced by these stressors, since the c-Abl inhibitor, Imatinib, blocked inactivation of parkin. Also, our finding that this pathway is seen predominantly in the striatum suggested that dopamine-containing cell types of the nigrostriatal pathway are particularly predisposed. c-Abl activation and tyrosine phosphorylation of parkin appear to reflect processes that are unique to the nigrostriatal pathway and not necessarily associated with inclusion bodies, since we did not observe c-Abl activation and tyrosine phosphorylation of parkin in the cortex, even in the four PD patients with neocortical Lewy bodies [14]. The c-Abl inhibitor, Imatinib, is a widely used chemotherapeutic agent for chronic myelogenous leukemia. We have shown that Imatinib inhibits c-Abl’s deleterious effects on parkin by preventing its phosphorylation and preserving its protective function, held promise for further testing of this agent as a neuroprotective therapeutic for PD. Since Imatinib has limited brain bioavailability [24], the amount of protection afforded by inhibition of c-Abl *in vivo* may be greatly improved by using related compounds with enhanced brain penetration, as inhibition of c-Abl in cellular models is profoundly protective.

INNO-406 is a second-generation tyrosine kinase inhibitor in development for treating Bcr-Abl+ leukemias, including chronic myelogenous leukemia (CML) and Philadelphia+ acute lymphoblastic leukemia. In preclinical studies, INNO-406 has been shown to be 25- to 55-fold more potent than Imatinib in vitro and ≥10-fold more potent in vivo [25]. A significant fraction of INNO-406 crosses the blood-brain barrier, reaching brain concentrations adequate for suppression of Bcr-Abl+ cells. Currently, INNO-406 is being developed in two phase II clinical trials for patients with B-cell chronic lymphocytic leukemia and prostate cancer, and a trial is in progress for patients with brain tumors [16], [26].

In our current studies, we show that INNO-406 significantly penetrates into the brain of mouse model of PD and prevents MPTP-induced activation of c-Abl and tyrosine phosphorylation of parkin. Furthermore, we show that INNO-406 very efficiently prevents dopaminergic neuronal and terminal damage and preserves dopamine content in the MPTP-mouse model of PD. Our results strengthen the data suggesting a role of parkin in the more common sporadic form of PD through oxidative stress, parkin phosphorylation, and the consequential loss of parkin ubiquitination and its protective function. Since oxidative stress is intricately involved in the activation of c-Abl and in sporadic PD, we propose that a novel, stress-induced cell signaling mechanism-involving activated c-Abl that inhibits parkin function and consequently increases the cell’s susceptibility to death due to accumulation of cytotoxic parkin substrates, such as AIMP2 is involved in this process. The evidence that INNO-406 mediated c-Abl inhibition and consequent inhibition of the phosphorylation of parkin, and the observation that restoration of parkin’s function is protective, suggests a promising approach for further assessment in the treatment of PD. Although we have previously shown that this c-Abl mediated damage was parkin dependent, in our current study we still can not rule out the fact that there might be some parkin/c-Abl independent pathway providing INNO-406 mediated neuroprotection. The elucidation of tyrosine phosphorylation-mediated inhibition of parkin activity and its pathological relevance as demonstrated in PD and models of PD will pave the way for a better understanding of the pathophysiology of this disease. Additionally, further studies in a post-MPTP treatment regimen of INNO-406 will be needed to further our significant preclinical findings to clinical translation.
